# Desmoplastic small round cell tumor (DSRCT) xenografts and tissue culture lines: Establishment and initial characterization

**DOI:** 10.3892/ol.2013.1265

**Published:** 2013-03-19

**Authors:** CONSTANTINE S.A. MARKIDES, DOUGLAS R. COIL, LINH H. LUONG, JOHN MENDOZA, TONY KOZIELSKI, DANA VARDEMAN, BEPPINO C. GIOVANELLA

**Affiliations:** Christus Stehlin Foundation for Cancer Research, Houston, TX 77025, USA

**Keywords:** desmoplastic small round cell tumor, sarcoma, xenograft, nude mice, tissue culture techniques

## Abstract

Desmoplastic small round cell tumor (DSRCT) is an extremely rare and aggressive neoplasm, which mainly affects young males and generally presents as a widely disseminated tumor within the peritoneal cavity. Due to the rarity of the tumor, its younger and overall healthier patient population (compared with other tumor types) and the fact that it lacks definitive histological and immunohistological features, the diagnosis of DSRCT may be frequently delayed or the tumor may be entirely misdiagnosed as a different type of abdominal sarcoma. The present study aimed to rectify the lack of models that exist for this rare neoplasm, through the development of several DSRCT tissue cultures and xenograft lines. Samples were received from surgeries and biopsies from patients worldwide and were immediately processed for xenograft development in nude mice. Tumor tissues were minced and fragments were injected into the dorsal flanks of nude mice. Of the 14 samples received, nine were established into xenograft lines and five into tissue culture lines. Xenografts displayed the microscopic histology of their parent tumors and demonstrated two different growth rates among the established xenograft lines. Overall, the establishment of these xenograft and tissue culture lines provides researchers with tools to evaluate DSRCT responses to chemotherapy and to investigate DSRCT-specific signaling pathways or mechanisms.

## Introduction

Desmoplastic small round cell tumor (DSRCT) is a rare and aggressive neoplasm that was first described by Gerald and Rosai (1). Although not unknown in females, DSRCT mainly affects young males and generally presents as a widely disseminated tumor within the peritoneal cavity. Other primary sites, including the paratesticular area, ovary, thorax, lung, and intracranial or head and neck areas, have also been documented (2–8). The majority of the literature regarding this particular type of tumor is comprised of case reports (9).

Clinically, DSRCT is usually diagnosed at an advanced stage, is highly aggressive and spreads along the peritoneum and mesothelial lined surfaces. Upon diagnosis, the tumor typically consists of a single large mass (occasionally as large as 40 cm in diameter) and multiple smaller masses scattered throughout the peritoneum, although other areas of origin have been noted. Common symptoms upon presentation include abdominal pain, hepatomegaly, ascites and hydronephrosis, and are non-specific and non-diagnostic (9). A definitive diagnosis is based on the identification of a reciprocal chromosome translocation, t(11;22)(p13;q12) (10). Due to the rarity of the tumor, its younger and overall healthier patient population (compared with other tumor types) and the fact that it lacks definitive histological and immunohistological features (9), DSRCT diagnosis may frequently be delayed or the tumor may be entirely misdiagnosed as a different type of abdominal sarcoma.

The scarcity of studies that are specific to DSRCT, as well as the non-specific presentation of the tumor, has resulted in difficulties regarding the treatment of the disease; according to the literature, no curative outcome has been achieved thus far (11). This is partly due to the fact that there are no available models that simulate the behavior of DSRCT outside the patient. The present study aimed to rectify this through the development of several DSRCT tissue cultures and xenograft lines.

## Materials and methods

### Patients and tissues

Given the low incidence of DSRCT, a limited number of patients were available for enrollment in the present study. Samples of tissues that had been removed during surgeries and biopsies, and that the local pathologists had confirmed as DSRCT, were received from surgical centers worldwide. Written informed consent was obtained from the patients for the use of their tissues in this study. The tissues were minced into small fragments (∼3 mm^3^) at the site of the surgery, and placed in sterile tubes containing RPMI-1640 medium supplemented with 10% fetal calf serum (FCS) and antibiotics (penicillin 50 ng/ml, streptomycin 50 ng/ml and neomycin 100 ng/ml). The tissue samples were shipped overnight on wet ice, although certain samples that were shipped from overseas took considerably longer than this to arrive. Upon arrival, the tumor samples were immediately processed for the development of xenograft lines. Following the establishment of the xenograft lines, several were transferred for use in cell cultures.

All portions of this study involving the use of laboratory animals were approved by the Institutional Animal Care and Use Committee (IACUC) of the CHRISTUS Stehlin Foundation for Cancer Research in Houston, Texas, USA. This committee operates under full compliance with OLAW (Office of laboratory animal welfare) regulations. All human tissues were obtained with full and proper consent from patients or their legal guardians.

### Xenograft establishment

The tissues were transferred to fresh, sterile phosphate-buffered saline (PBS) solution and rinsed. They were then further minced to a final fragment size of <1 mm^3^ using crossed scalpels or sharp iris scissors. The fragments were then transferred using a wide-tip pipette to a 15- or 50-ml sterile centrifuge tube or a universal container, and centrifuged gently for 2 min. The supernatant was removed and replaced with RPMI-1640 medium to a volume equal to that of the tumor. Following mixing, the suspended tumor fragments were injected subcutaneously with a 16-gauge needle into the dorsal flanks of non-inbred Swiss nude mice (nu^−^/nu^−^), and allowed to grow. The mice were inspected daily for a minimum of six months. When growth was detected, the volume of the tumor was determined by measurement of the three main diameters with calipers. The tumors were harvested and passaged once their volume reached ∼4,000 mm^3^. Following every passage, each tumor was studied by protein gel electrophoresis for allozymic differences at two enzyme loci. The xenografts were also examined histologically to confirm that the morphological characteristics of DSRCT had been retained through the establishment process. Once established, each xenograft or cell culture tumor line was assigned a unique three letter code used for identification, e.g., BER or ZUC.

### Cell culture

There was no single procedure utilized for all specimens. An initial gross examination of the tumor specimen assisted the determination of the appropriate following steps. A general procedure involved mincing the tumor fragments as finely as possible using sharp, sterile iris scissors. The tumor specimen was then washed extensively in PBS solution and transferred to a flask that had been pretreated with collagen. Fresh DMEM/F12 medium supplemented with 10% FCS was added to the flask and attachments to the flask surface were facilitated for 18–24 h at 37°C. The medium was replaced no more than 24 h after the cells were first placed in the flask. This procedure removed any unattached or necrotic tissue that may have disrupted the growth of the attached fragments. The growth medium was replaced regularly (at least weekly, but more often if necessary) in the first flask until the outgrowth had spread to cover 50% of the growth surface, at which point the cells were passaged. Fibroblastic growth was culled from the cultures slowly over the course of the experiment (≥6 months) by careful, selective trypsinization.

## Results

### Morphology

**Figure 1:**
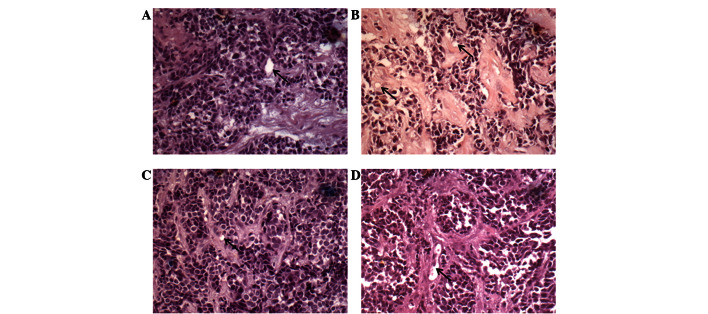
Histology of desmoplastic small round cell tumor (DSRCT) xenografts. Four separate passages of the same DSRCT (BER cell line) as a xenograft in nude mice. Tumor samples were received on wet ice from biopsies or surgery, and were processed as described in the Materials and methods. The nude passages (NPs) depicted are (A) NP0, (B) NP3, (C) NP6 and (D) NP9. All passages display the characteristic desmoplastic recruitment of fibroblasts, while the tumor cells are small with large nuclei and little cytosol. Vascularization (marked with black arrows) is primarily evident within the desmoplastic tissue, as opposed to penetrating the tumor cell clusters. Hematoxylin and eosin staining; magnification, ×200.

Upon surgical removal and macroscopic observation, the DSRCT xenografts were soft to the touch and appeared to be hypervascularized and flushed with blood. Microscopically, the xenografts were similar in appearance to metastatic tumors isolated from patients. As demonstrated in Fig. 1, the tumors consisted of small compact cells, arranged in clusters of varying sizes and surrounded by desmoplastic stromal tissues, which were primarily fibroblastic in nature. The quantity of stromal tissue varied from field to field, even within the same xenograft passage. The tumor cells were small with large, round, hyperchromatic nuclei. Little cytosol was observed and the cell borders were typically indistinct. Heavy vascularization was evident in the stromal tissue, while the majority of the core small cell sections of the tumor were lacking in any significant vascular recruitment.

In several instances, the tissue received from the patient sites was either dry, frozen solid or unrefrigerated. No growth was predicted or observed in these samples. However, even under these circumstances, the tissues were implanted into the mice and treated in the same way as the remaining tissues that had been received, in case any cells had survived.

Of the 14 human DSRCT specimens that were received in good condition and implanted into the nude mice, nine (64%) were considered to be positive for growth. These tumor lines were assigned the code names; ZUC, BER, VOS, MYE, BOD, UEK, ORA, DYC and CAR. In all positive cases, the site of inoculation grew a tumor that was histologically compatible (Fig. 1) with the original tissue, and this tumor was able to be retransplanted into other nude mice for serial passaging. For the nine established xenograft lines, the mean time to the first passage (nude passage 1, NP1) was 192 days (Table I). The time to the first passage is an effective measure of tumor malignancy, i.e., how well the tumor adapts to an environment other than its tissue of origin.

Attempts to inject the tumor fragments intraperitoneally were conducted in order to simulate orthotopic inoculation; however, for reasons that are presently unclear, these attempts were universally unsuccessful at generating tumors in the nude mice.

**Figure 2:**
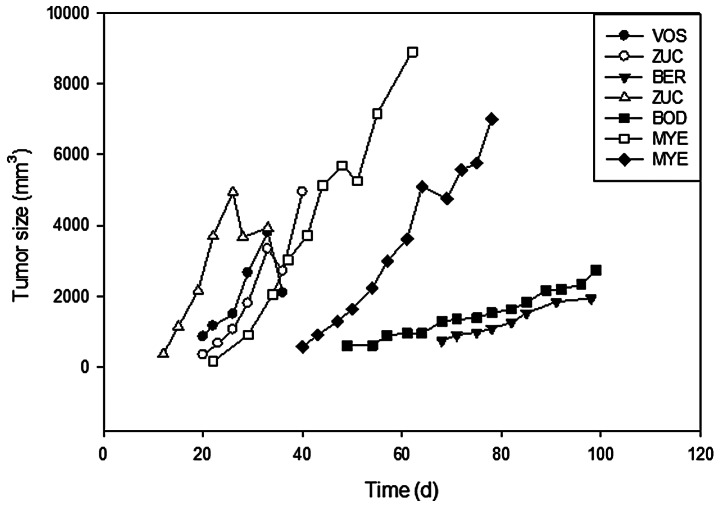
Growth rate of established desmoplastic small round cell tumor (DSRCT) xenografts. Tumor fragments were injected subcutaneously into the dorsal flank of nude mice on day 0. Tumors were measured in three dimensions with calipers, twice weekly, to determine the tumor volume. Two distinct populations are visible: a rapid growth group (ZUC, VOS, MYE) and a slow growth group (BOD, BER). Duplicate entries of the same tumor are represented by separate growth curves of the successive passages of the xenografts.

When cell lines become established, either as xenografts or in tissue culture, their growth characteristics may markedly change compared with those of primary inoculations of patient tissues in nude mice. The growth rates of the established xenograft lines were separated into two categories: a rapid growth group and an indolent growth group (Fig. 2). The mean time from tumor injection to palpable growth (200–300 mm^3^) was 22.4 days for the rapid growth group and 58 days for the indolent growth group.

Of the few samples that yielded sufficient tissue to split between the xenograft lines and tissue cultures, none yielded primary lines in tissue culture. However, several lines were able to be established as nude transfer (NUT) lines, which were xenografts that were surgically excised from nude mice and subsequently grown in culture. The stromal tissue was culled slowly from these cultures by selective trypsinization over the course of the experiment (≥6 months).

## Discussion

Histologically, the DSRCT xenografts closely resembled the tumors from which they originated; they appeared to be small compact cells, with large nuclei and little cytoplasm, arranged in clusters threaded with desmoplastic tissue. The simplicity of the cell structure, combined with the multifocal nature of the disease in patients, suggested the possibility that an unusually large proportion of the tumor cells may be cancer stem cells.

When excised from the mice, the xenografts were observed to be soft, well-vascularized and flushed with blood. The majority of other dorsally grown subcutaneous xenografts exhibit a necrotic core that has been starved of nutrients by the lack of sufficient vascularization. In numerous types of solid tumor, particularly those of the breast, desmoplasia is initiated by the secretion of platelet-derived growth factor (PDGF), which acts in a paracrine manner on stromal fibroblasts (12). The same mechanism is likely to be involved in DSRCT, while the dense vascularization of the stromal tissues closely resembles the situation in a number of types of colon carcinoma, where the recruited fibroblasts contribute to vascular maturation (13).

While the present data indicate two distinct populations of DSRCT as far as growth rate is concerned, the underlying mechansims for these differences are unclear. Traditional cytotoxic agents are more effective against fast-growing tumors, and slower-growing tumors are less responsive to cell cycle phase-specific cytotoxic agents (14). Therefore, we predicted that the DSRCT lines that exhibited more indolent growth would be less susceptible to cell cycle-specific chemotherapy, such as with camptothecins or methotrexate. Limited testing in the present study suggested that this, in fact, was not the case.

DSRCT belongs to a larger family of tumors known as small round cell sarcomas. Neoplasms, including Wilms’ tumor and Ewing’s sarcoma, also belong to this category. DSRCT is significantly less common than the remaining tumors of the family, but have the worst prognosis. DSRCTs have been demonstrated to be refractory to chemotherapy and almost uniformly fatal (9), however, the tumors may respond well to camptothecin compounds, which have been identified to have a certain effectiveness against indolent tumors (15,16).

## Figures and Tables

**Table I:** Growth of primary DSRCT samples as xenografts in nude mice.

Tumor	Date received	Time to NP1 (days)
ZUC	01/28/2005	145
BER	02/11/2005	75
VOS	04/15/2005	271
MYE	10/04/2005	113
BOD	11/18/2006	186
UEK	01/11/2007	209
ORA	05/23/2008	306
DYC	02/13/2009	250
CAR	05/18/2010	177

Time to first passage is a measure for the malignancy of the tumor, i.e. how likely it is to grow in a tissue different than that of its origin. For the nine established xenograft lines, the mean time to the first passage [nude passage 1 (NP1)] was 192 days. DSRCT, desmoplastic small round cell tumor.

## Abbreviations:

DSRCT: desmoplastic small round cell tumor
FCS: fetal calf serum
PDGF: platelet derived growth factor
